# 2-Oxo-2-phenyl­ethyl benzoate

**DOI:** 10.1107/S1600536811018976

**Published:** 2011-05-25

**Authors:** Hoong-Kun Fun, Suhana Arshad, B. Garudachari, Arun M. Isloor, M. N. Satyanarayan

**Affiliations:** a X-ray Crystallography Unit, School of Physics, Universiti Sains Malaysia, 11800 USM, Penang, Malaysia; b Organic Chemistry Division, Department of Chemistry, National Institute of Technology-Karnataka, Surathkal, Mangalore 575 025, India; c Department of Physics, National Institute of Technology-Karnataka, Surathkal, Mangalore 575 025, India

## Abstract

In the title compound, C_15_H_12_O_3_, the terminal phenyl rings make a dihedral angle of 86.09 (9)° with each other. In the crystal, a pair of inter­molecular C—H⋯O hydrogen bonds link the mol­ecules, forming a dimer with an *R*_2_^2^(10) ring motif.

## Related literature

For background to and applications of phenacyl benzoates, see: Huang *et al.* (1996 ▶); Gandhi *et al.* (1995 ▶); Ruzicka *et al.* (2002 ▶); Litera *et al.* (2006 ▶); Sheehan & Umezaw (1973 ▶). For bond-length data, see: Allen *et al.* (1987 ▶). For hydrogen-bond motifs, see: Bernstein *et al.* (1995 ▶).
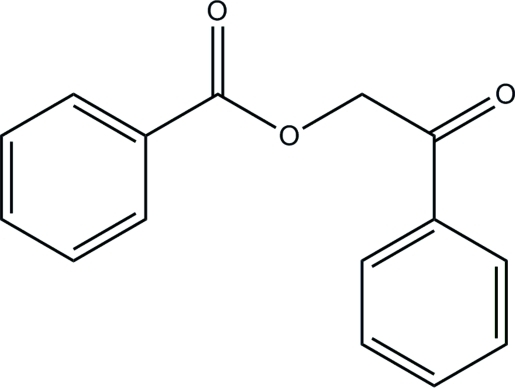


## Experimental

### 

#### Crystal data

C_15_H_12_O_3_
*M* *_r_* = 240.25
Monoclinic, 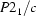

*a* = 9.0299 (13) Å
*b* = 14.116 (2) Å
*c* = 9.6379 (14) Å
β = 90.564 (3)°
*V* = 1228.4 (3) Å^3^
*Z* = 4
Mo *K*α radiation
μ = 0.09 mm^−1^
*T* = 296 K
0.77 × 0.52 × 0.43 mm

#### Data collection

Bruker SMART APEXII DUO CCD area-detector diffractometer
Absorption correction: multi-scan (*SADABS*; Bruker, 2009 ▶) *T*_min_ = 0.934, *T*_max_ = 0.963
23225 measured reflections
3573 independent reflections
2408 reflections with *I* > 2σ(*I*)
*R*_int_ = 0.035

#### Refinement

*R*[*F* ^2^ > 2σ(*F* ^2^)] = 0.060
*wR*(*F* ^2^) = 0.192
*S* = 1.05
3573 reflections
163 parameters
H-atom parameters constrained
Δρ_max_ = 0.25 e Å^−3^
Δρ_min_ = −0.19 e Å^−3^

### 

Data collection: *APEX2* (Bruker, 2009 ▶); cell refinement: *SAINT* (Bruker, 2009 ▶); data reduction: *SAINT*; program(s) used to solve structure: *SHELXTL* (Sheldrick, 2008 ▶); program(s) used to refine structure: *SHELXTL*; molecular graphics: *SHELXTL*; software used to prepare material for publication: *SHELXTL* and *PLATON* (Spek, 2009 ▶).

## Supplementary Material

Crystal structure: contains datablocks global, I. DOI: 10.1107/S1600536811018976/is2715sup1.cif

Structure factors: contains datablocks I. DOI: 10.1107/S1600536811018976/is2715Isup2.hkl

Supplementary material file. DOI: 10.1107/S1600536811018976/is2715Isup3.cml

Additional supplementary materials:  crystallographic information; 3D view; checkCIF report

## Figures and Tables

**Table 1:** Hydrogen-bond geometry (Å, °)

*D*—H⋯*A*	*D*—H	H⋯*A*	*D*⋯*A*	*D*—H⋯*A*
C8—H8*B*⋯O3^i^	0.97	2.57	3.454 (2)	152

Symmetry code: (i) 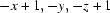.

**Fig. 1.**
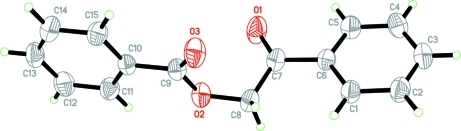
The molecular structure of the title compound, showing 30% probability displacement ellipsoids and the atom-numbering scheme.

**Fig. 2.**
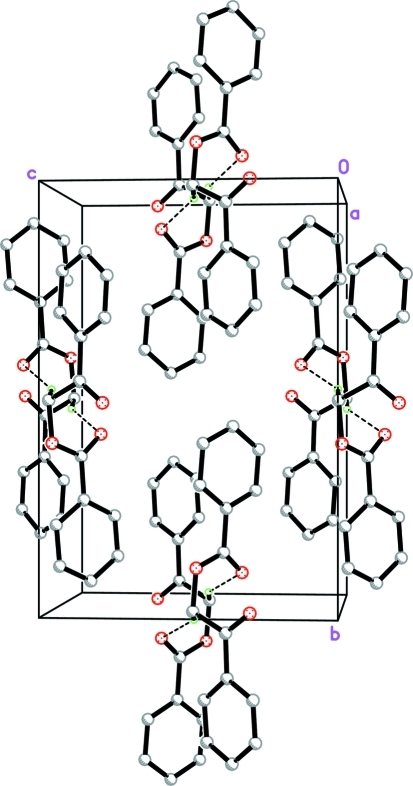
The crystal packing of the title compound. Dashed lines represent the hydrogen bonds.

## supplementary crystallographic information

### Comment

In organic chemistry, phenacyl benzoate is a derivative of an acid, formed by reaction between acid and phenacyl bromide. They find applications in the field of synthetic chemistry (Huang *et al.*, 1996; Gandhi *et al.*, 1995) such as synthesis of oxazoles, imidazoles, benzoxazepines. They are also useful for photo-removable protecting groups for carboxylic acids in organic synthesis and biochemistry (Ruzicka *et al.*, 2002; Litera *et al.*, 2006; Sheehan & Umezaw, 1973). Keeping this in view, the title compound was synthesized to study its crystal structure.

The molecular structure is shown in Fig. 1. The terminal phenyl rings (C1–C6 and C10–C15) make a dihedral angle of 86.09 (9)° with each other. Bond lengths (Allen *et al.*, 1987) and angles are within normal range. In the crystal packing (Fig. 2), pairs of intermolecular C8—H8B···O3 hydrogen bonds (Table 1) link the molecules to form dimers, generating *R*^2^_2_(10) ring motifs (Bernstein *et al.*, 1995).

### Experimental

The mixture of benzoic acid (1.0 g, 0.008 mol), sodium carbonate (0.95 g, 0.009 mol) and 2-bromo-1-phenylethanon (1.7 g, 0.009 mol) in dimethyl formamide (10 ml) was stirred at room temperature for 2 h. On cooling, the separated colourless needle-shaped crystals of 2-oxo-2-phenylethyl benzoate were collected by filtration. Compound was recrystallized from ethanol (yield: 1.91 g, 97.4%; *m.p*: 390–391 K).

### Refinement

All H atoms were positioned geometrically (C—H = 0.93 or 0.97 Å) and refined using a riding model with *U*_iso_(H) = 1.2*U*_eq_(C).

### Crystal data

**Table:**

C_15_H_12_O_3_	*F*(000) = 504
*M**_r_* = 240.25	*D*_x_ = 1.299 Mg m^−^^3^
Monoclinic, *P*2_1_/*c*	Mo *K*α radiation, λ = 0.71073 Å
Hall symbol: -P 2ybc	Cell parameters from 6650 reflections
*a* = 9.0299 (13) Å	θ = 2.6–29.6°
*b* = 14.116 (2) Å	µ = 0.09 mm^−^^1^
*c* = 9.6379 (14) Å	*T* = 296 K
β = 90.564 (3)°	Block, colourless
*V* = 1228.4 (3) Å^3^	0.77 × 0.52 × 0.43 mm
*Z* = 4	

### Data collection

**Table:**

Bruker SMART APEXII DUO CCD area-detector diffractometer	3573 independent reflections
Radiation source: fine-focus sealed tube	2408 reflections with *I* > 2σ(*I*)
graphite	*R*_int_ = 0.035
φ and ω scans	θ_max_ = 30.1°, θ_min_ = 2.3°
Absorption correction: multi-scan (*SADABS*; Bruker, 2009)	*h* = −12→12
*T*_min_ = 0.934, *T*_max_ = 0.963	*k* = −19→19
23225 measured reflections	*l* = −13→13

### Refinement

**Table:**

Refinement on *F*^2^	Primary atom site location: structure-invariant direct methods
Least-squares matrix: full	Secondary atom site location: difference Fourier map
*R*[*F*^2^ > 2σ(*F*^2^)] = 0.060	Hydrogen site location: inferred from neighbouring sites
*wR*(*F*^2^) = 0.192	H-atom parameters constrained
*S* = 1.05	*w* = 1/[σ^2^(*F*_o_^2^) + (0.0845*P*)^2^ + 0.258*P*] where *P* = (*F*_o_^2^ + 2*F*_c_^2^)/3
3573 reflections	(Δ/σ)_max_ < 0.001
163 parameters	Δρ_max_ = 0.25 e Å^−^^3^
0 restraints	Δρ_min_ = −0.19 e Å^−^^3^

### Special details

**Table:**

Geometry. All e.s.d.'s (except the e.s.d. in the dihedral angle between two l.s. planes) are estimated using the full covariance matrix. The cell e.s.d.'s are taken into account individually in the estimation of e.s.d.'s in distances, angles and torsion angles; correlations between e.s.d.'s in cell parameters are only used when they are defined by crystal symmetry. An approximate (isotropic) treatment of cell e.s.d.'s is used for estimating e.s.d.'s involving l.s. planes.
Refinement. Refinement of *F*^2^ against ALL reflections. The weighted *R*-factor *wR* and goodness of fit *S* are based on *F*^2^, conventional *R*-factors *R* are based on *F*, with *F* set to zero for negative *F*^2^. The threshold expression of *F*^2^ > σ(*F*^2^) is used only for calculating *R*-factors(gt) *etc*. and is not relevant to the choice of reflections for refinement. *R*-factors based on *F*^2^ are statistically about twice as large as those based on *F*, and *R*- factors based on ALL data will be even larger.

### Fractional atomic coordinates and isotropic or equivalent isotropic displacement parameters (Å^2^)

**Table:**

	*x*	*y*	*z*	*U*_iso_*/*U*_eq_	
O1	0.89801 (19)	0.00806 (10)	0.7007 (2)	0.0993 (6)	
O2	0.75397 (14)	0.10379 (9)	0.50588 (15)	0.0704 (4)	
O3	0.56279 (16)	0.08421 (9)	0.64940 (17)	0.0813 (4)	
C1	0.7648 (2)	−0.20439 (12)	0.5344 (2)	0.0647 (4)	
H1A	0.6994	−0.1779	0.4702	0.078*	
C2	0.7776 (3)	−0.30148 (14)	0.5449 (2)	0.0790 (6)	
H2A	0.7197	−0.3403	0.4884	0.095*	
C3	0.8752 (2)	−0.34132 (13)	0.6386 (2)	0.0751 (5)	
H3A	0.8843	−0.4068	0.6443	0.090*	
C4	0.9593 (2)	−0.28413 (15)	0.7237 (2)	0.0762 (5)	
H4A	1.0248	−0.3110	0.7876	0.091*	
C5	0.9467 (2)	−0.18735 (13)	0.7146 (2)	0.0693 (5)	
H5A	1.0037	−0.1490	0.7726	0.083*	
C6	0.84954 (16)	−0.14632 (11)	0.61946 (17)	0.0544 (4)	
C7	0.84060 (17)	−0.04164 (12)	0.6139 (2)	0.0602 (4)	
C8	0.7618 (2)	0.00214 (13)	0.4948 (2)	0.0642 (4)	
H8A	0.8123	−0.0145	0.4098	0.077*	
H8B	0.6621	−0.0233	0.4891	0.077*	
C9	0.64689 (17)	0.13613 (11)	0.59154 (17)	0.0548 (4)	
C10	0.64216 (16)	0.24087 (11)	0.60075 (17)	0.0524 (4)	
C11	0.7258 (2)	0.29780 (13)	0.5158 (2)	0.0693 (5)	
H11A	0.7899	0.2708	0.4520	0.083*	
C12	0.7138 (3)	0.39614 (14)	0.5260 (3)	0.0826 (6)	
H12A	0.7692	0.4350	0.4683	0.099*	
C13	0.6201 (2)	0.43540 (13)	0.6216 (2)	0.0772 (6)	
H13A	0.6122	0.5009	0.6284	0.093*	
C14	0.5385 (2)	0.37905 (14)	0.7064 (2)	0.0760 (5)	
H14A	0.4760	0.4063	0.7713	0.091*	
C15	0.5483 (2)	0.28147 (13)	0.6964 (2)	0.0659 (5)	
H15A	0.4919	0.2432	0.7540	0.079*	

### Atomic displacement parameters (Å^2^)

**Table:**

	*U*^11^	*U*^22^	*U*^33^	*U*^12^	*U*^13^	*U*^23^
O1	0.1077 (11)	0.0599 (8)	0.1294 (13)	0.0017 (8)	−0.0530 (10)	−0.0200 (8)
O2	0.0689 (8)	0.0530 (7)	0.0896 (9)	0.0013 (5)	0.0084 (7)	0.0022 (6)
O3	0.0744 (8)	0.0530 (7)	0.1171 (12)	−0.0103 (6)	0.0231 (8)	0.0051 (7)
C1	0.0653 (10)	0.0523 (9)	0.0764 (11)	−0.0002 (7)	−0.0094 (8)	−0.0014 (8)
C2	0.0890 (14)	0.0553 (10)	0.0924 (14)	−0.0088 (9)	−0.0098 (11)	−0.0055 (9)
C3	0.0767 (12)	0.0514 (9)	0.0975 (14)	0.0015 (8)	0.0076 (10)	0.0079 (9)
C4	0.0682 (11)	0.0654 (11)	0.0948 (14)	0.0087 (9)	−0.0041 (10)	0.0141 (10)
C5	0.0603 (10)	0.0634 (11)	0.0841 (12)	0.0002 (8)	−0.0104 (9)	0.0002 (9)
C6	0.0460 (7)	0.0506 (8)	0.0668 (9)	0.0018 (6)	0.0047 (6)	−0.0028 (7)
C7	0.0478 (8)	0.0516 (8)	0.0811 (11)	0.0003 (6)	−0.0023 (7)	−0.0079 (8)
C8	0.0620 (9)	0.0524 (9)	0.0780 (11)	0.0042 (7)	−0.0026 (8)	−0.0127 (8)
C9	0.0485 (8)	0.0484 (8)	0.0675 (9)	−0.0030 (6)	−0.0033 (7)	0.0011 (7)
C10	0.0481 (7)	0.0459 (8)	0.0629 (9)	−0.0010 (6)	−0.0074 (6)	0.0018 (6)
C11	0.0770 (12)	0.0556 (10)	0.0753 (11)	−0.0033 (8)	0.0097 (9)	0.0039 (8)
C12	0.1004 (15)	0.0544 (10)	0.0930 (14)	−0.0110 (10)	0.0040 (12)	0.0145 (10)
C13	0.0865 (13)	0.0464 (9)	0.0985 (15)	0.0037 (9)	−0.0135 (11)	−0.0027 (9)
C14	0.0737 (12)	0.0592 (10)	0.0953 (14)	0.0071 (9)	0.0016 (10)	−0.0154 (10)
C15	0.0623 (10)	0.0577 (9)	0.0776 (11)	−0.0023 (8)	0.0048 (8)	−0.0036 (8)

### Geometric parameters (Å, °)

**Table:**

O1—C7	1.205 (2)	C7—C8	1.481 (3)
O2—C9	1.357 (2)	C8—H8A	0.9700
O2—C8	1.441 (2)	C8—H8B	0.9700
O3—C9	1.197 (2)	C9—C10	1.482 (2)
C1—C2	1.379 (3)	C10—C11	1.378 (2)
C1—C6	1.385 (2)	C10—C15	1.383 (2)
C1—H1A	0.9300	C11—C12	1.396 (3)
C2—C3	1.376 (3)	C11—H11A	0.9300
C2—H2A	0.9300	C12—C13	1.374 (3)
C3—C4	1.375 (3)	C12—H12A	0.9300
C3—H3A	0.9300	C13—C14	1.362 (3)
C4—C5	1.374 (3)	C13—H13A	0.9300
C4—H4A	0.9300	C14—C15	1.384 (3)
C5—C6	1.389 (2)	C14—H14A	0.9300
C5—H5A	0.9300	C15—H15A	0.9300
C6—C7	1.481 (2)		
			
C9—O2—C8	114.58 (13)	O2—C8—H8B	109.1
C2—C1—C6	119.97 (17)	C7—C8—H8B	109.1
C2—C1—H1A	120.0	H8A—C8—H8B	107.9
C6—C1—H1A	120.0	O3—C9—O2	122.46 (15)
C3—C2—C1	120.47 (19)	O3—C9—C10	124.34 (15)
C3—C2—H2A	119.8	O2—C9—C10	113.16 (13)
C1—C2—H2A	119.8	C11—C10—C15	119.85 (16)
C4—C3—C2	119.89 (18)	C11—C10—C9	121.99 (15)
C4—C3—H3A	120.1	C15—C10—C9	118.15 (15)
C2—C3—H3A	120.1	C10—C11—C12	119.62 (19)
C5—C4—C3	120.06 (19)	C10—C11—H11A	120.2
C5—C4—H4A	120.0	C12—C11—H11A	120.2
C3—C4—H4A	120.0	C13—C12—C11	119.83 (19)
C4—C5—C6	120.55 (18)	C13—C12—H12A	120.1
C4—C5—H5A	119.7	C11—C12—H12A	120.1
C6—C5—H5A	119.7	C14—C13—C12	120.46 (18)
C1—C6—C5	119.05 (16)	C14—C13—H13A	119.8
C1—C6—C7	122.68 (15)	C12—C13—H13A	119.8
C5—C6—C7	118.27 (15)	C13—C14—C15	120.29 (19)
O1—C7—C8	119.72 (16)	C13—C14—H14A	119.9
O1—C7—C6	122.22 (17)	C15—C14—H14A	119.9
C8—C7—C6	118.04 (14)	C10—C15—C14	119.94 (18)
O2—C8—C7	112.43 (14)	C10—C15—H15A	120.0
O2—C8—H8A	109.1	C14—C15—H15A	120.0
C7—C8—H8A	109.1		
			
C6—C1—C2—C3	−0.7 (3)	C8—O2—C9—O3	−2.3 (2)
C1—C2—C3—C4	0.9 (3)	C8—O2—C9—C10	179.89 (14)
C2—C3—C4—C5	−0.5 (3)	O3—C9—C10—C11	−169.63 (18)
C3—C4—C5—C6	−0.2 (3)	O2—C9—C10—C11	8.1 (2)
C2—C1—C6—C5	0.0 (3)	O3—C9—C10—C15	9.2 (3)
C2—C1—C6—C7	−179.66 (17)	O2—C9—C10—C15	−173.09 (14)
C4—C5—C6—C1	0.4 (3)	C15—C10—C11—C12	−0.7 (3)
C4—C5—C6—C7	−179.90 (18)	C9—C10—C11—C12	178.10 (18)
C1—C6—C7—O1	169.12 (19)	C10—C11—C12—C13	0.7 (3)
C5—C6—C7—O1	−10.6 (3)	C11—C12—C13—C14	−0.1 (3)
C1—C6—C7—C8	−12.7 (2)	C12—C13—C14—C15	−0.6 (3)
C5—C6—C7—C8	167.58 (16)	C11—C10—C15—C14	0.1 (3)
C9—O2—C8—C7	−79.71 (18)	C9—C10—C15—C14	−178.74 (16)
O1—C7—C8—O2	−5.0 (2)	C13—C14—C15—C10	0.5 (3)
C6—C7—C8—O2	176.76 (14)		

### Hydrogen-bond geometry (Å, °)

**Table:**

*D*—H···*A*	*D*—H	H···*A*	*D*···*A*	*D*—H···*A*
C8—H8B···O3^i^	0.97	2.57	3.454 (2)	152

Symmetry codes: (i) −*x*+1, −*y*, −*z*+1.
